# A benchmark analysis of feature selection and machine learning methods for environmental metabarcoding datasets

**DOI:** 10.1016/j.csbj.2025.04.017

**Published:** 2025-04-16

**Authors:** Erik Zschaubitz, Henning Schröder, Conor Christopher Glackin, Lukas Vogel, Matthias Labrenz, Theodor Sperlea

**Affiliations:** a Department of Biological Oceanography, Leibniz Institute for Baltic Sea Research, Seestraße 15, Rostock, 18119, Germany; b Planet AI GmbH, Warnowufer 60, Rostock, 18057, Germany

**Keywords:** Microbial ecology, Machine learning, Feature selection, Benchmark, Metabarcoding, Framework

## Abstract

Next-Generation Sequencing methods like DNA metabarcoding enable the generation of large community composition datasets and have grown instrumental in many branches of ecology in recent years. However, the sparsity, compositionality, and high dimensionality of metabarcoding datasets pose challenges in data analysis. In theory, feature selection methods improve the analyzability of eDNA metabarcoding datasets by identifying a subset of informative taxa that are relevant for a certain task and discarding those that are redundant or irrelevant. However, general guidelines on selecting a feature selection method for application to a given setting are lacking. Here, we report a comparison of feature selection methods in a supervised machine learning setup across 13 environmental metabarcoding datasets with differing characteristics. We evaluate workflows that consist of data preprocessing, feature selection and a machine learning model by their ability to capture the ecological relationship between the microbial community composition and environmental parameters. Our results demonstrate that, while the optimal feature selection approach depends on dataset characteristics, feature selection is more likely to impair model performance than to improve it for tree ensemble models like Random Forests. Furthermore, our results show that calculating relative counts impairs model performance, which suggests that novel methods to combat the compositionality of metabarcoding data are required.

## Highlights

• Comparison of feature selection and ML models on 13 metabarcoding datasets.
• Random Forest models excel in regression and classification tasks.
• Recursive Feature Elimination enhances Random Forest performance across various tasks.
• Ensemble models are robust without feature selection in high-dimensional data.
• Open-source framework for customizable metabarcoding data analysis.

## Graphical abstract

Our benchmark comparison of feature selection methods in machine learning workflows for environmental microbiome datasets suggests that Random Forests without additional feature selection generally perform well. Created in BioRender.

**Figure:**
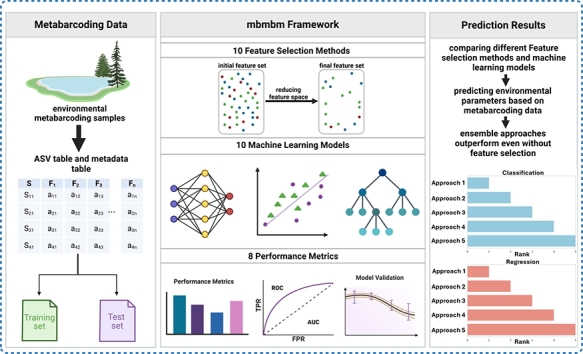

## 1 Introduction

The rapid expansion of human activities and industrialization profoundly impacts ecosystems globally, leading to accelerated biodiversity loss, habitat degradation, and climate change progression [1], [2], [3]. Understanding how anthropogenic disturbances impact ecosystem dynamics is critical for sustainable environmental management [4]. In recent years, the combination of high-throughput DNA sequencing methods and machine learning (ML) or artificial intelligence methods has received heightened attention in the context of biomonitoring [5], [6]. This is because next-generation sequencing methods such as metabarcoding can capture a snapshot of the biodiversity of a broad taxonomic range of organisms in a noninvasive and automatable manner by targeting fingerprint-like genetic marker regions like the 16S rDNA gene (for prokaryotes), 18S rDNA gene (for eukaryotes, broadly), cytochrome oxidase 1 (for animals) or the ITS region (for fungi). Metabarcoding of environmental water samples often results in thousands to hundred of thousands of so-called Operational Taxonomic Units (OTUs) or Amplicon Sequencing Variants (ASVs), i.e., abstract and technically defined groups of organisms that do not conform to taxonomic nomenclature. Whereas OTUs represent sequences that are clustered according to a similarity threshold, ASVs are exact sequence variants distinguished after accounting for potential sequencing errors [7], [8].

Supervised ML encompasses data analysis methods that can capture associations between a set of input features (i.e., in this case, OTUs or ASVs) and a target variable. In learning, ML approaches implicitly distinguish between relevant patterns and noise (such as contamination, technical variation or ecological processes irrelevant to the task at hand) and by testing the models on held-out datasets, we can make sure that the captured associations hold. Furthermore, many ML approaches account for nonlinear relationships among input features and between input features and the target variable. This property is important for the study of ecosystems, as we expect there to be interactions between species that modulate their respective responses to external stimuli in a non-linear manner [9]. Taken together, supervised ML and metabarcoding facilitate the use of morphologically indiscriminate microbes as bioindicators of environmental states or levels of potential pollution [10], [11], [12], [13], [14], [15], [16], [17].

The analysis of environmental metabarcoding datasets is complicated by their sparsity, compositionality, and, more often than not, a mismatch between the large numbers of detected organisms and the number of samples that are gathered [9], [18]. The latter issue, which is known as the curse of dimensionality, can lead to a loss of efficiency, speed, accuracy, and interpretability in data analysis with increasing numbers of features [19], and it is further aggravated by the nonlinearity and sparsity of metabarcoding datasets [9]. A solution to this problem can be found in the form of dimensionality reduction (DR) and feature selection (FS) methods. Whereas the former transforms the data into new, smaller, and more expedient feature spaces, the latter selects features with relevance to the analysis task at hand while discarding others. In the context of ML workflows, DR and FS can be further classified relative to the model as follows: Filter methods select features prior to passing to the model. Wrapper methods use the model to select features, and embedded methods are integrated with the model (for more details, see Materials and Methods) [20].

There is no consensus on which FS method is optimal for metabarcoding data sets. Neither should we expect there to be a “one size fits all” FS method because they quantify feature relevance in different ways [21]. Comparative studies have demonstrated that which FS method is optimal for a given dataset depends on the characteristics of the dataset and task at hand [22], [23]. This has been observed across various areas of computational biology, including the analysis of microarray data [24], gene expression profiles [25], clinical datasets [26], fish distributions [27] and quantitative structure-activity relationships [28]. Differences in dataset characteristics derive not only from differences in study object or biological variations between datasets, but also differences in the way that raw sequencing data is preprocessed into OTU or ASV tables, including, e.g. post-clustering tools like lulu, mumu or dbOTU3 [29], [30], [31]. Nevertheless, it should be possible to provide general advice on the choice of FS methods based on a large-scale benchmark comparison. A survey of FS methods for the environmental metabarcoding dataset is currently lacking.

In this paper, we present a benchmark comparison of filter, wrapper, and embedded FS methods in regression and classification settings based on 13 publicly available large microbial metabarcoding sequence datasets in a ML workflow. We focus on microbial datasets because these have been the focus of recent interest in sequence-based, ML-powered biomonitoring [5], [6] but show that our results also hold for metabarcoding datasets of fish populations. Guided by the idea that the composition of the biota in an ecosystem reflects the abiotic state of the ecosystem, we evaluate and compare ML models with regard to their ability to approximate the latter from the former. To facilitate our benchmark comparison, we developed a Python package that wraps available FS methods and implements methods that have not been available in Python until now. The Microbiome Machine Learning Benchmark (mbmbm) framework is highly modular, easily customizable, and available for public use (https://github.com/erikzsch/mbmbm).

We identify FS methods that allow ML models to achieve high prediction performances in a short runtime. Although our results support the notion that the optimal FS method depends on the dataset and task, we are able to demonstrate that tree ensemble models, such as Random Forest (RF) and Gradient Boosting (GB), consistently outperform other approaches independent of FS method, due to their ability to model high-dimensional, nonlinear relationships. FS methods like recursive feature elimination (RFE) and variance thresholding (VT), can further enhance the performance of RF and GB, with VT significantly reducing runtime by eliminating low-variance features. However, many FS methods inadvertently discard relevant OTUs or ASVs, emphasizing the robustness of RF and GB models without FS. Additionally, the compositional nature of the sequencing data significantly affects model performance. Models trained on absolute ASV or OTU counts outperformed those using relative counts, likely because normalization obscures important ecological patterns. Although linear FS methods, such as Pearson and Spearman correlation, perform better on relative counts, they are generally less effective than nonlinear methods like mutual information (MI) or FS-free tree ensemble models.

## 2 Material and methods

### 2.1 Datasets

As the basis of this benchmark study, we selected publicly available metabarcoding datasets that contain large numbers of samples and were created using an internally uniform methodology (Table 1). To be able to make generalizable statements from the results of this benchmark study, datasets were chosen to show a high heterogeneity between each other in terms of habitat type and sampling area. Furthermore, whenever possible, ASV or OTU tables were downloaded in processed form to account for variance introduced by differences in sequence analysis pipelines, including whether the data contains OTUs or ASVs. See Table S1 for weblinks to the sources of the ASV and metadata tables. Target variables used in this study were required to not contain missing values. The choice of target variable was based on an expectation that there is a correlation between the microbiome and the variable at hand, often underpinned by results published with the respective dataset. Due to a small number of categorical target variables in the metadata of all datasets, numerical targets with clear and distinct levels were chosen as classification target for some datasets (such as atl_ocean_transect, bog_lakes, bedford_basin_V4V5, and bedford_ basin_V6V8).

**Table 1:** Overview of the datasets used in the benchmark experiment. The target variables in front of the slash were used in the regression task, and variables after the slash were used in the classification task. Dashed lines in the target variable column indicate that no classification task was performed for the dataset. The boso_fish dataset was used to show that the results gathered for microbial community datasets transfer to multicellular communities.

Data set name	Description	Target region	# Samples	# features (ASVs/ OTUs)	ASV or OTU	Target variable	Ref.
atl_ocean_transect	Atlantic water samples	16S rRNA (V4-V5)	113	765	OTU (99%)	depth/depth	[36]
Australia	Coastal water, Australia	16S (V1-V3), 18S (V4)	2620	16383	zOTU (unoise3)	phosphate/-	[37]
bedford_basin_V4V5	Bedford Basin water	16S (V4-V5)	753	490	ASV (deblur)	pressure/year	[38]
bedford_basin_V6V8	Bedford Basin water	16S (V6-V8)	693	378	ASV (deblur)	pressure/year	[38]
bog_lakes	8 bog lakes, N. Wisconsin	16S (V4)	790	6902	ASV (deblur)	temp/Depth	[39]
eu_lakes_allfeatures	255 European lakes	16S (V2-V3)	86	315733	OTU (swarm)	Mg/-	[40], [41], [42]
eu_lakes_allsamples	255 European lakes	16S (V2-V3)	232	315733	OTU (swarm)	pH/-	[40], [41], [42]
nz_springs	925 geothermal springs, NZ	16S (V4)	923	32659	OTU (USEARCH)	turbidity/-	[43]
ports	Global port water samples	16S (V4-V5)	601	117398	ASV (DADA2)	salinity/-	[44], [45]
subseafloor_arc	Global subseafloor	16S rRNA	299	7926	OTU (USEARCH)	water depth/aerobicity	[46]
subseafloor_bac	Global subseafloor	16S rRNA	299	35642	OTU (USEARCH)	sediment depth/aerobicity	[46]
tara_oceans	Ocean water globally	16S rRNA	139	35652	mOTU (USEARCH)	depth/biome type	[47]
wastewater_treatment	Sludge from 269 WWTPs, 23 countries	16S (V4)	687	66743	OTU (UPARSE)	avg. temp/Continent	[48]

boso_fish	Seawater samples from Boso peninsula	12S (mito-chondrial)	530	856	ASV (unoise3)	avg. water_tem, salinity/site_name	[49]

Rarefaction curves were generated using the function *rarefaction_curve* from the vegan package (v2.6-6, [32]), and Bray-Curtis NMDS plots were produced with the *metaMDS* function (using *distance= “bray”*) from vegan to identify samples that were outliers based on sequence counts or their relative similarity to other samples. Outlier samples were removed from further analysis. Only samples with both metadata and community composition data were retained, and metadata variables with minimal missing values were selected. ASVs or OTUs with zero variance and control samples were excluded; controls were not used to control for contamination. The OTU and ASV tables were preprocessed by imputing missing values with zero counts. Metadata, ASV tables, and taxonomy data were preprocessed into a unified tabular format. Data files were imported using functions from readxl (v1.4.3, [33]), *OTUtable* (v1.1.2, [39]), and *data.table* (v1.15.4, [34]). Data wrangling was performed using functions from dplyr (v1.1.4, [35]).

### 2.2 Feature selection methods

In this study, we employed three primary types of FS methods: filter, wrapper, and embedded methods. Each of these approaches provides unique advantages and they are explored in greater depth in the following sections. The specific FS methods compared in this benchmarking study are easily accessible in e.g. Python packages or well-used in the field (for a list, see Table 2).

**Table 2:** The feature selection methods used in this benchmark. Methods used for classification tasks are indicated with a “c”, methods used for regression are marked with “r” in the “task” column. ^1^ Combined with univariate functions. ^2^ Code adopted from https://github.com/SantiagoEG/FCBF_module/blob/master/FCBF_module.py.

FS method	Task	Hyperparameter setting	Source
None	c/r	-	
IndVal	c/r	*percentile* = 50	[54]
		*num*_*permutations* = 20	
SelectFromModel	c/r		sklearn
Recursive Feature	c/r	*n*_*features*_*to*_*select* = 100	sklearn
Elimination		*step* = 0.05	
Generic Univariate	c/r ^1^	*mode* = *k*_*best*	sklearn
Select		*param* = 100	
Fast Correlation-	c/r	*threshold* = 0.01	[51]^2^
Based Filter			

Univariate functions			

Chi squared	c/		sklearn
ANOVA F-value	c/		sklearn
Mutual information	c/r		sklearn
Variance threshold	c/r	*threshold* = 0.8 (absolute counts);	sklearn
		*threshold* = 0.001 (relative counts)	
Pearson's r	/r		sklearn
F-statistic	/r		sklearn

#### 2.2.1 Filter methods

Univariate filter methods typically assign a measure of importance f(x,y) regarding the target variable *y* to each feature x∈X, and then select features based on a cutoff threshold. The variance threshold filter, a simple example of this method, selects features by removing those with variance below a specified threshold, calculated as follows:

(1) $$\frac{1}{n}\sum_{i=1}^{N}{\left(x_{i}-\overset{¯}{X}\right)}^{2},$$

where *N* is the number of samples to remove features that do not change and $\overset{¯}{X}$ is the mean of all values of feature X, and $x_{i}$ is the value of feature X for the i-th sample.

The Pearson correlation filter selects features according to their linear relationship to the target variable as follows:

(2) $$\frac{\sum_{i=1}^{N}(x_{i}-\overset{¯}{x})(y_{i}-\overset{¯}{y})}{\sqrt{\sum_{i=1}^{N}{\left(x_{i}-\overset{¯}{x}\right)}^{2}\sum_{i=1}^{N}{\left(y_{i}-\overset{¯}{y}\right)}^{2}}}.$$

Here, $x_{i}$ represents the value of feature *x* for the *i*-th sample, and $\overset{¯}{x}$ denotes the mean of all values of the feature *x*. Similarly, $y_{i}$ is the value of the variable *y* in the *i*-th sample, while $\overset{¯}{y}$ denotes the mean of all values of the variable *y*.

For categorical target variables with *k* categories or levels, the chi-squared filter selects features that deviate most from the null hypothesis distribution under the assumption of independence between *x* and *y*. This is calculated using the following equation:

(3) $$\sum_{k}^{K}\frac{{\left(\sum_{i}^{N}x_{i}(y_{i}=k)-\sum_{i}^{N}x_{i}p(k)\right)}^{2}}{\sum_{i}^{N}x_{i}p(k)},$$

where p(k) is the ratio of samples belonging to the category *k*.

Along similar lines, the mutual information filter selects features related to the target variable in information-theoretical terms, measuring the amount of information shared between each feature and the target. The mutual information quantifies the reduction in uncertainty of the target variable given the knowledge of a feature and is estimated using a nearest-neighbor method [50], but is ultimately calculated as.:

(4) $$MI(x,y)=\sum_{x_{i}\in x}\sum_{y_{j}\in y}p(x_{i},y_{j})\log\left(\frac{p(x_{i},y_{j})}{p(x_{i})p(y_{j})}\right),$$

The F-value ANOVA filter evaluates the ratio of the variability of the features between groups determined by the continuous target variable and the variance within these groups. It is calculated using:

(5) $$\frac{\frac{1}{K-1}\sum_{i=k}^{K}n_{k}{\left(\overset{¯}{x_{k}}-\overset{¯}{x}\right)}^{2}}{\frac{1}{n-K}\sum_{k=1}^{K}\sum_{i=1}^{n_{k}}{\left(x_{ki}-\overset{¯}{x_{k}}\right)}^{2}},$$

where *K* is the number of categories in the target variable, $n_{k}$ is the number of samples in category *k*, $\overset{¯}{x}$ and ${\overset{¯}{x}}_{k}$ are the mean value of *x* in all samples and all samples belonging to category *k*, respectively, and $x_{ki}$ is the *i*th sample in category *k*.

The Indicator Value (IndVal) method is a tool for identifying bioindicators for groups of sites that are widely used in ecology. For each species (or, more general, feature) *i* and each group of sites (or level of the categorical target variable) *k*, the value is assigned as follows:

(6) $$\frac{n_{ik}}{n_{i}}\times \frac{C_{ik}}{C_{i}},$$

where the first term specifies the specificity of $x_{i}$ for site group *k*, i.e., the relative amount of species $x_{i}$ present in samples from site group *k*, and the second term defines the fidelity, i.e., the fraction of sites at which species $x_{i}$ are present and that also belong to site group *k*. This function was implemented in Python in the framework for the first time.

In addition to these univariate filter methods, we also employed the multivariate Fast Correlation-Based Filter (FCBF), which selects features that are both related to the target and representative of groups of similar features [51]. To begin, the concept of entropy is initially defined to quantify the uncertainty or randomness in the distribution of a variable or feature. For a distinct variable or feature *X*, the entropy is given by:

(7) $$H(X)=-\sum_{x\in X}p(x)\log p(x),$$

where p(x) denotes the probability of each value *x* in *X*. Higher entropy indicates greater unpredictability (e.g., a uniform distribution), whereas lower entropy indicates greater certainty (e.g., a single dominant value). Using this concept, the symmetrical uncertainty (SU) is computed as the mutual information *MI* between each feature and the target variable, normalized by the sum of the entropies *H* of both:

(8) $$SU(x,y)=2\times \frac{MI(x,y)}{H(x)+H(y)},$$

FCBF retains only features whose SU exceeds a predefined threshold. Then, it iteratively calculates the SU between the remaining features to remove redundant features, ensuring that only features that are not related to each other are retained.

#### 2.2.2 Wrapper methods

Wrapper methods integrate ML model training and testing in the search for the optimal feature subset. Although wrapper methods produce feature subsets that optimally fit the model, recurrent model training processes lead to much longer runtimes than filter methods, especially for large feature spaces. We use one wrapper method in this benchmark, RFE, which recursively removes the least important features based on model performance until the desired number of features is achieved [52]. The initial ML model is trained on all features. Then, features are ranked according to their feature importance or model feature coefficients, the least important features are pruned from the set, and a new model is trained. This process is repeated recursively, making RFE particularly effective at reducing overfitting by eliminating less relevant features. This, in turn, enhances model generalizability, especially in high-dimensional datasets. Additionally, RFE allows flexibility in choosing the base estimator (e.g., linear models, decision trees) to match the model requirements and domain-specific needs, further refining FS for performance and interpretability.

#### 2.2.3 Embedded methods

Embedded methods fully integrate FS into the model construction process. The models in this study that incorporate embedded FS methods include RF [53] and lasso regression. RF trees are ensemble decision trees trained on random subsets of the features of the dataset. Only the feature that minimizes a decision metric at is used at every split in the decision trees making this an implicit FS method. The most popular choices for the decision metric are the variance given by

(9) $$Var(Y)=\frac{1}{2|Y|}\sum_{i}^{\left|Y\right|}\sum_{j}^{\left|Y\right|}{\left(y_{i}-y_{j}\right)}^{2}$$

and the Gini impurity given by

(10) $$Gini(Y)=1-\sum_{y\in Y}p{\left(y\right)}^{2},$$

where *Y* is the set of the values of the target variable and |Y| is its cardinality, for regression and classification problems, respectively. Furthermore, the selective splitting process reduces model complexity and computational cost by focusing only on informative features, thus enhancing model interpretability and robustness. In addition, feature importance scores from RFs provide insights into the relative importance of each feature across the ensemble, which makes them useful for feature ranking.

Lasso regression is a modification of linear regression. The proposed method minimizes the residual sum of squares with a constraint on the sum of the absolute values of the coefficients, i.e., its fitness function becomes

(11) $$\min\sum_{1}^{\left|Y\right|}|y_{i}-\overset{ˆ}{y}{|}^{2}+\lambda \sum_{1}^{n}|\beta_{i}|,$$

where $\overset{ˆ}{y_{i}}$ is the value the model predicted for instance *i*, *β* represent the model coefficients and *λ* is a parameter controlling regularization strength. This modification causes some coefficients to be zero, thereby implicitly performing FS. Lasso's embedded FS is particularly advantageous in high-dimensional datasets because it simplifies the model and enhance interpretability by effectively isolating key predictors.

### 2.3 Supervised model training and validation

ML models used in this study are listed in Table 3. These models were chosen to cover the space of ML strategies evenly and were used with default hyperparameter settings unless otherwise noted in Table 3. No hyperparameter optimization was performed. Deep learning models are not covered by this benchmark comparison due to their high training data requirement. For model training and evaluation, the datasets were split into 80% for training and 20% for testing. The performance of the ML models was validated using appropriate metrics for both classification and regression tasks. For classification, the following metrics were employed in a multiclass setting: F1 score, recall, precision, accuracy, and area under receiver operating characteristic curve (AU-ROC). For the regression tasks, we used the mean square error (MSE), Mean Absolute Error (MAE), and coefficient of determination ($R^{2}$). All performance metrics were implemented using the TorchMetrics library [55].

**Table 3:** ML models used in this benchmark. Models above the middle line were used for classification, and models below the middle line were used for regression. Hyperparameter settings are only reported if they depart from default settings.

Model name	Sklearn function	Hyperparameter
AdaBoost	ensemble.AdaBoostClassifier	
Naive Bayes	naive_bayes.GaussianNB	
Neural Network	neural_network.MLPClassifier	
Random Forest	ensemble.RandomForestClassifier	*max*_*depth* = 5
Support Vector Classifier	svm.SVC	*C* = 0.025

AdaBoost	ensemble.AdaBoostRegressor	
Lasso Regression	linear_model.Lasso	
Linear Regression	linear_model.LinearRegression	
Support Vector Regression	svm.SVR	
Random Forest	ensemble.RandomForestRegressor	

### 2.4 The mbmbm framework

To ensure parallelized, repeatable, and well-documented training and evaluation of models with different hyperparameter settings, we developed a Python-based Microbiome Machine Learning Benchmark (mbmbm) framework. In it, the steps in a ML workflow, such as data loading, preprocessing of target variables and features, FS, dimensionality reduction, model choice, and model evaluation, are formalized in classes, providing basic programming interfaces that enable high modularity and build on abstractions used in the scikit-learn package [56]. Thus, the mbmbm framework is adaptable and easily extendable with new methods, enabling efficient benchmark comparisons as demonstrated in this paper. Furthermore, we incorporated checkpoints after each step in the ML workflow. This approach facilitates incremental adjustments for different dataset characteristics, enabling faster retraining or tuning of specific sections without re-running the entire pipeline. In the mbmbm framework, complete workflows containing all hyperparameter settings can be either specified in Python code or using YAML-based configuration files via Hydra [57]. The mbmbm framework logs processes using loguru (v0.7.0, [58]) and tracks the runtime, applies FS, train models, and evaluates results using metrics from TorchMetrics [55], saving evaluations and total runtime. Preliminary visualizations can be generated to guide further model selection. The complete mbmbm framework can be installed using poetry (v1.5.1, [59]).

The benchmark calculations presented here were conducted in Python 3.11 using the Python packages numpy (v1.23.4, [60]), matplotlib (v3.6.2, [61]), pandas (v1.5.2, [62]), torch (v2.0, [63]), scikit-learn (v1.2.2, [56]), loguru (v0.7.0, [58]), hydra-core (v1.3.2, [57]), and skorch (v1.0.0, [64]). All computations were executed on a high-performance system equipped with two AMD EPYC 9534 processors and 1152 GiB ECC DDR-5 memory, which enabled efficient parallel execution of the benchmarks. However, the proposed framework is also suitable for single desktop PCs, thereby making it accessible to various computational resources.

### 2.5 ML workflow evaluation and statistical ranking

In ML workflows, the interplay between workflow elements impacts prediction performance and runtime efficiency. To determine the optimal FS and ML methods for metabarcoding datasets, we conducted an exhaustive grid search across preprocessing, FS, and ML methods using the models, FS techniques, and datasets listed in Table 1, Table 2, Table 3, respectively, with the default hyperparameters used in scikit-learn, unless otherwise listed there. No further parameter tuning was performed. Both classification and regression tasks were performed because these usually require very different ML models and FS techniques and the outcomes of benchmark comparisons might, therefore, diverge drastically.

The complexity of the relationship between metabarcoding and environmental data can vary between target variables and datasets. To control for these differences when comparing model performances and runtimes, we ranked each model's results for each target feature from best (e.g., highest $R^{2}$, highest multiclass F1 score, or lowest runtime) to worst (e.g., lowest $R^{2}$, lowest multiclass F1 score, or highest runtime). The ranks achieved by each methodological approach were then averaged across datasets. To determine the significance of the differences in performance of two approaches, we performed pairwise Wilcoxon rank sum tests on the ranks assigned to each approach and applied false discovery rate (FDR) correction to the p-values for regression and classification tasks separately. Finally, as metabarcoding data is compositional, we compared the performance of the models on the absolute OTU or ASV counts to datasets transformed to relative abundances. This is one of the main preprocessing methods used for combatting compositionality; others, such as centered log-ratio transforms or rarefaction lead to prohibitive runtimes in preliminary tests.

In this paper, the discussion of results focuses exclusively on the ($R^{2}$) for regression tasks and F1 score for classification tasks to streamline the interpretation and comparison of model performance, but further performance metrics and partial runtimes have been calculated and are present in Table S2. Additionally, only the total runtime of the models and the model performance on the validation datasets is reported. Model performance ranking and visualization were conducted outside the mbmbm framework using the packages reshape2 (v1.4.4, [65]), patchwork (v1.2.0, [66]), and ggplot2 (v3.5.1, [67]) in R (v4.4.1).

## 3 Results and discussion

### 3.1 Performance analysis of regression models

To compare the performance of FS methods in regression tasks, we used the mbmbm framework and analysed the results separately on (1) absolute ASV counts and (2) ASV counts converted to relative abundances, normalizing each sample to account for the compositional nature of metabarcoding data. A third option to combat compositionality, repeatedly rarefying and then analysing the samples, was not used because with the repetitions necessary to overcome random effects in rarefaction, runtimes for large datasets and slow FS methods become prohibitively long.

**Fig. 1:**
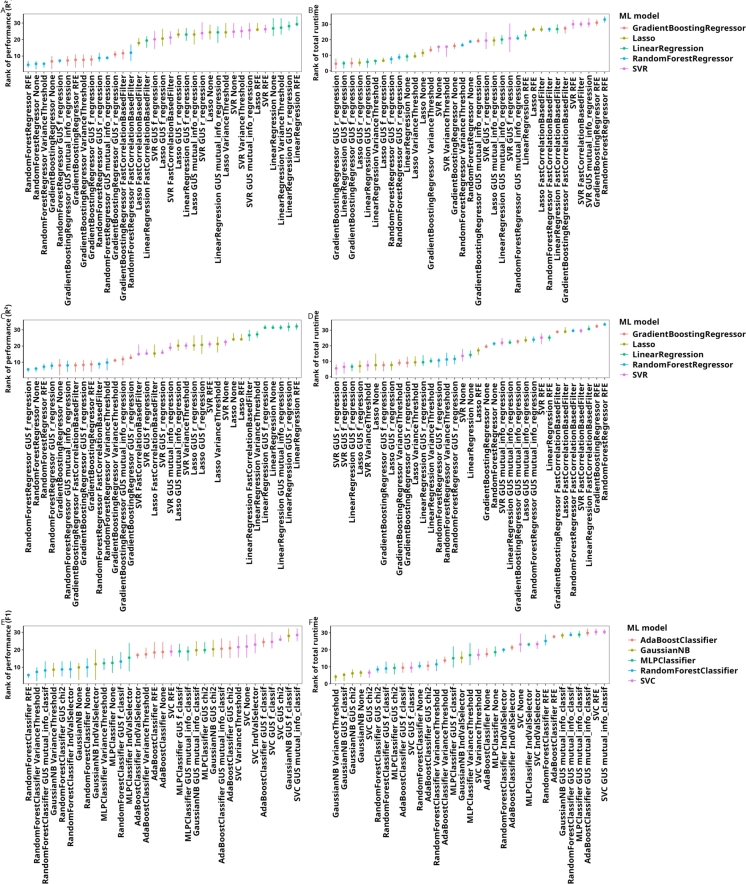
Results of the benchmark comparison for regression and classification tasks, aggregated across the datasets by ranking. In all subfigures, points represent the average rank and lines represent the interquartile range of the ranks attributed to each approach. For all rankings, lower ranks represent better results. Ranked performances (A, C, E) and ranked total runtimes (B, D, F); Regression on absolute ASV counts (A, B), regression on relative ASV counts (C, D), and classification on absolute counts (E, F). Abbreviations: GUS (Generic Univariate Select), FCBF (Fast Correlation-Based Filter).

**Fig. 2:**
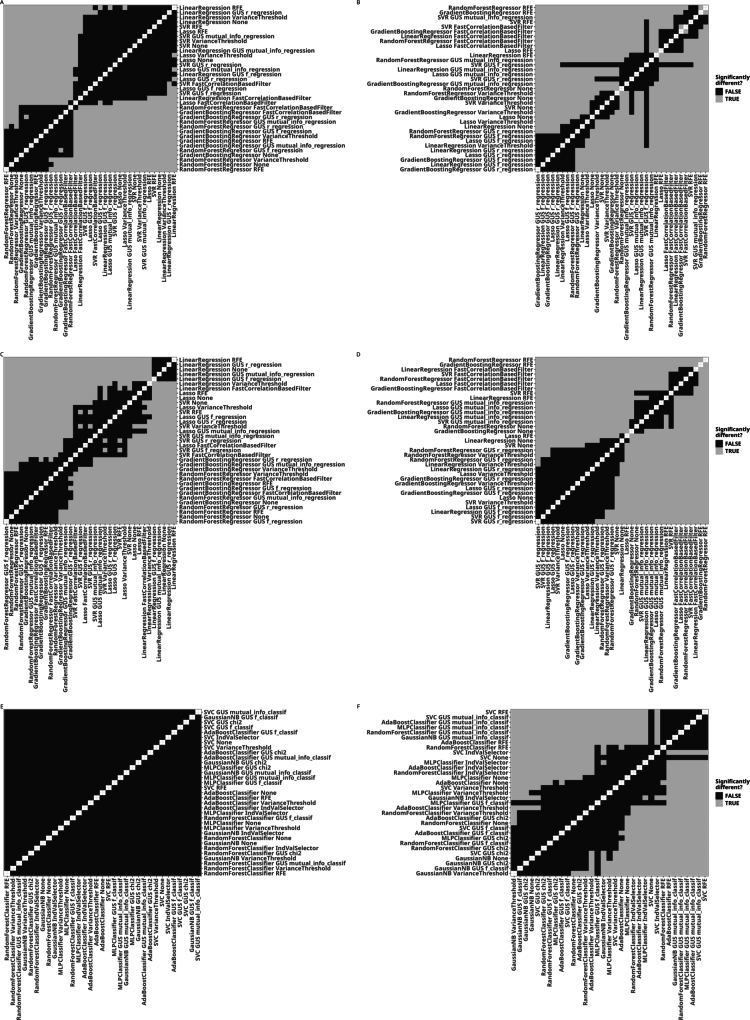
Significant and insignificant differences in the rankings of different approaches derived using a Wilcoxon rank sum test after FDR for the performance (A, C, E) and runtime (B, D, F) of the approaches displayed in Fig. 1 to solve the regression task on absolute (A, B) and relative ASV counts (C, D) and the classification task (E, F). The axis labels are sorted by the average rank in the respective figure in Fig. 1. All p values are available in the Table S3.

For both the absolute and the relative case, RF and GB models consistently outperformed the other models (Fig. 1A and 1C; full results in Table S2). When using absolute ASV counts, the top three approaches involved either RFE, VT, or no FS method. Furthermore, approaches involving RF and GB models formed a group of insignifically different performances that are, however, significantly different from the approaches not involving tree ensemble models (Fig. 2A and 2C).

The total runtimes of the approaches examined in this study ranged from 0.05 seconds to 37.11 hours, with 70% of the approaches having a total runtime of less than a minute (see Table S2). The VT filter reduced runtime compared to no FS, whereas RFE exhibited the longest runtimes across all datasets (Fig. 1B). The result suggests that removing low-variance features can reduce the training time of RF models, whereas the recursive nature of RFE leads to considerable increases in runtime. While the choice of hyperparameter values influences the runtime of the FS method, by, for example, reducing the numbers of recursion for RFE, we expect there to be a payoff between runtime and model quality. As the approaches with the longest runtimes consistently include RFE and FCBF and these methods have longer runtimes on larger datasets, the choice of faster methods is adviced for large-scale studies if a high-performance computing system is unavailable.

### 3.2 Effects of absolute and relative ASV counts on model performance

**Fig. 3:**
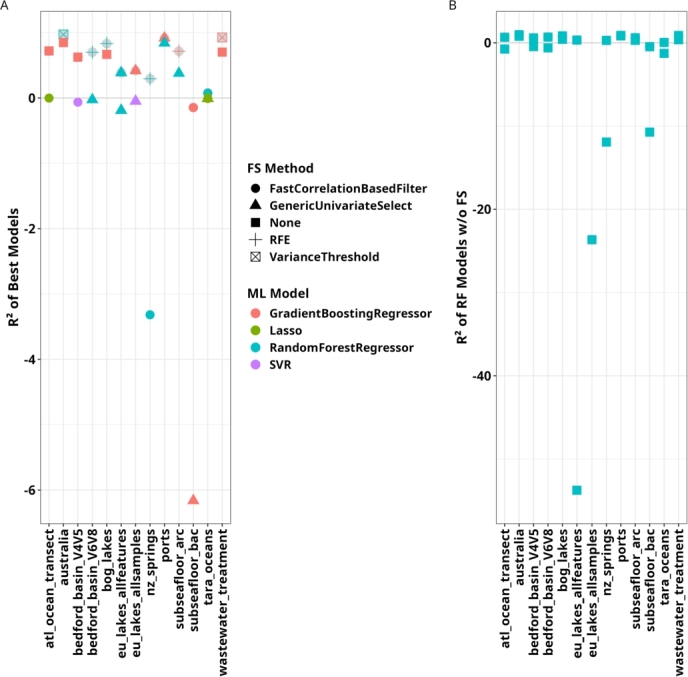
Comparison of benchmark results between absolute and relative ASV counts. Results for absolute ASV counts are highlighted using grey circles. For each dataset, we show (A) the approach with the best result and (B) the result for the RF model without FS.

When using relative ASV abundances, FS methods based on Pearson and Spearman correlation, which are designed to capture linear relationships, rank among the best-performing approaches (Fig. 1C). This is in notable contrast to our results on absolute ASV counts and suggests that the relationships between microbial community composition and the chosen target parameters may exhibit more linear characteristics when relative counts are used for the former. However, linear regression models themselves perform poorly, emphasizing the necessity of nonlinear ML models to accurately capture complex ecological interactions. Thus, instead of linear FS methods increasing in performance on relative counts, these results might be due to non-linear FS methods decreasing model performance when run on relative counts. In addition, models trained and tested on relative ASV counts consistently underperformed as compared to those using absolute ASV counts (Fig. 3). This results suggests that the absolute counts might contain ecologically relevant information that is lost in normalization. Taken together, these findings underscore the importance of FS methods capable of leveraging both linear and nonlinear patterns, i.e., model flexibility to adapt to a broad range of data characteristics within ecological datasets.

### 3.3 Dataset-specific feature selection and model performance

**Fig. 4:**
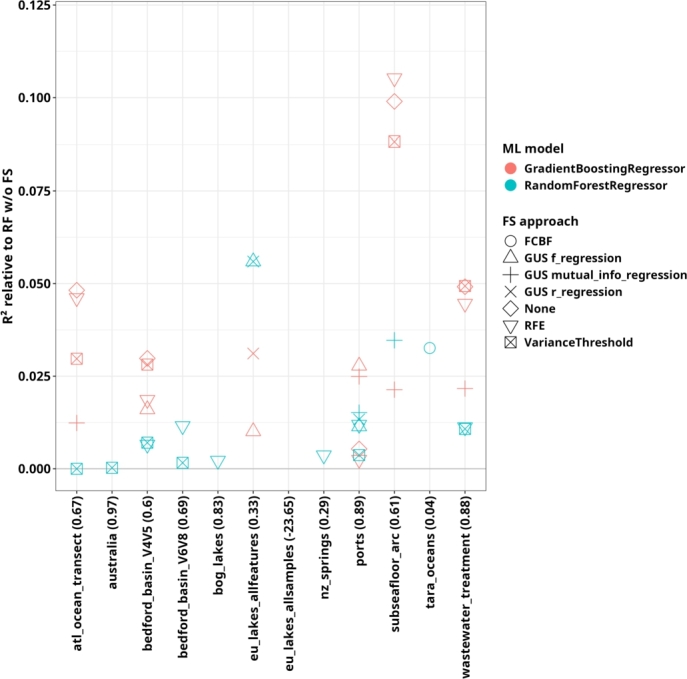
Difference between the *R*^2^ scores of the approaches that outperformed the RF without FS and the RF without FS for the regression task on absolute ASV counts. The numbers in brackets after the dataset name indicate the *R*^2^ score of the RF without FS. Only models outperforming the RF and showing a performance with a positive *R*^2^ values are shown.

A ranked model comparison, like the one presented in the preceding paragraphs, may obscure dataset-specific performance differences. To address these differences, we focus on approaches that outperform the baseline RF model without FS on specific datasets (Fig. 4). As anticipated from the general ranking results (Fig. 1A), the group of approaches that surpass the RF without FS primarily consists of GB and RF models, typically combined with RFE, VT, or no FS method. In specific datasets, such as wastewater_treatment, subseafloor_arc, and atl_ocean_transect, we observed that models employing MI as a univariate FS method outperform the RF without FS, which suggests that MI is particularly valuable for capturing nonlinear relationships in these contexts. Conversely, for datasets like ports, eu_lakes_allfeatures, and bedford_basin_V4V5, approaches using linear FS methods based on Pearson or Spearman correlation also exceed the performance of the RF without FS. These results underscore that the relationship between ASVs and target variables influences the relative effectiveness of different FS methods. Especially, for datasets with more linear relationships, regression-based univariate FS methods are beneficial, whereas MI-based methods are better suited for identifying features relevant to RF and GB models when relationships are more categorical or complex. Taken together, these results underscore the importance of choosing FS methods that align with the data structure and target variable nature to optimize predictive performance.

### 3.4 Feature selection and model performance in classification tasks

Comparing the performance of ML approaches and FS methods in classification tasks yields results similar to those observed for regression tasks (Fig. 1E). The top three approaches utilize an RF model combined with either RFE, VT, or MI as the FS method. However, unlike in regression tasks, the RF without FS ranks lower and is outperformed by approaches that incorporate Gaussian naive Bayes models. The difference underscores that FS and ML model effectiveness can vary significantly between regression and classification tasks. The variance in ranking of the approaches is higher in classification than in regression, making statistical comparisons across approaches less informative (Fig. 2). This variability may indicate that classification tasks are more sensitive to dataset-specific characteristics and model-parameter interactions, which can impact consistency across methods. Notably, classification-specific FS methods such as MI, chi-squared (chi2), and IndVal are ranked better than regression-focused FS methods. Classification tasks require discriminating between values whereas, in regression tasks, FS methods identify continuous relationships, pointing to differences in the strategy that FS methods require in regression and classification tasks. The observed outcomes emphasize the need to align FS methods with task-specific requirements to achieve optimal classification model performance. Specifically, our benchmark comparison indicates that tree ensemble models, such as RF and GB, generally perform well across tasks—without FS for regression tasks, with variance-based filters or RFE for both regression and classification, or with classification-specific filters like MI, for categorical outcomes (Fig. 1).

### 3.5 Applications of tree-based models for environmental metabarcoding data

**Fig. 5:**
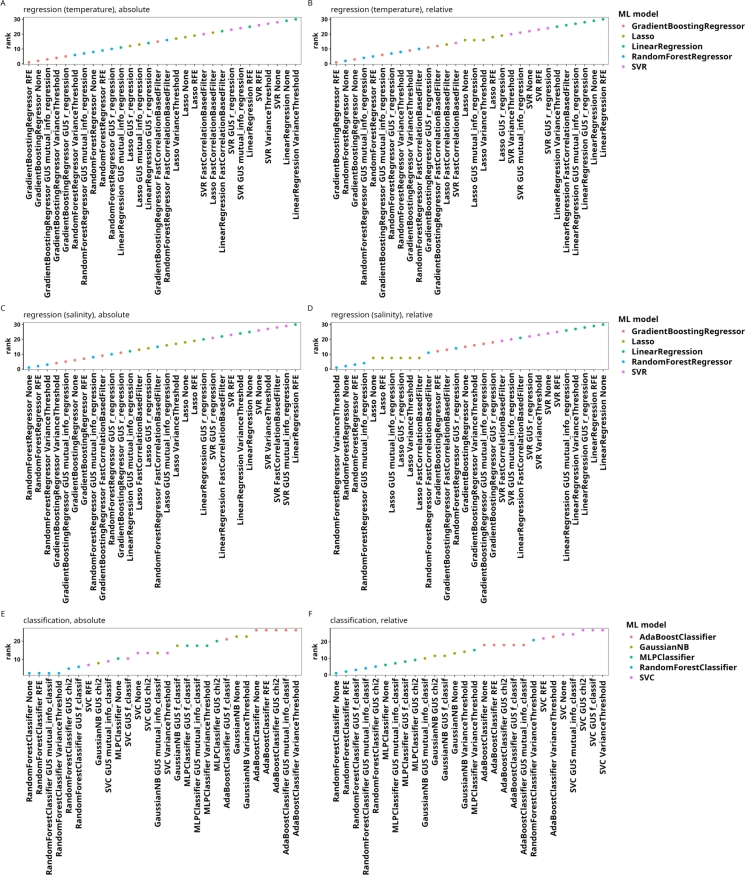
Ranked results of the benchmark comparison for regression (A-D) and classification (E, F) tasks on absolute (A, C, E) and relative counts (B, D, F) for the boso_fish dataset, which surveys the fish communities around the boso peninsula. The classification target is the sampling location. Abbreviations: GUS (Generic Univariate Select), FCBF (Fast Correlation-Based Filter).

The high performance of RF and GB models in the benchmark comparison presented here is not surprising given their capability to model complex, nonlinear relationships between features and target variables, and interactions among features, in high-dimensional datasets [53], [68]. Their versatility makes them well-suited to metabarcoding datasets, where complex ecological relationships often require flexible modeling. Additionally, these findings align with previous results from the application of ML methods in ecology [12], [16], [69], [70], [71], [72], [73], [74], [75], [76] as well as more general studies on tabular data [77], [78], reinforcing the value of tree ensemble methods for varied and high-dimensional data. To test whether the results we gained up to this point mostly targetting the environmental microbiome generalize to other parts of the biota, we also analysed a dataset describing the fish community surrounding the Boso peninsula in Japan choosing sampling location, water temperature and water salinity as target parameters (Fig. 5). For either target parameter, RF and GBR models without FS or with FS based on RFE, VT or MI outperform the other models, supporting the generality of our findings. Because other high-dimensional, sequencing-based datasets such as metagenomics and metatranscriptomics datasets share the basic characteristics that make RF and GB models fitting for the analysis of metabarcoding datasets, we expect a high relative performance of these models for those datasets as well.

Unlike many other tabular datasets, sequencing data are inherently compositional, meaning that the values of individual features (e.g., ASV counts) are constrained by a constant total, which violates the assumptions of many standard data analysis methods [79], [80]. In the context of ML, we hypothesized that using relative rather than absolute ASV counts might address the challenges associated with compositionality, potentially leading to improved model performance [81]. However, the observed decline in ML model performance when using relative ASV counts compared to absolute counts suggests that sequencing depth does indeed reflect the ecological state of the ecosystems sampled instead of purely representing a technical artifact. (Fig. 3). Another method for controlling for differences in sampling effort, rarefaction, involves randomly subsampling samples to the same sequencing depth. Aside from its questionable admissibility in data analyses aside from calculating alpha diversities [82], [83], its inherent randomness makes a high number of repetitions necessary, which leads to prohibitively long runtimes in ML workflows.

Notably, for both the regression task on absolute counts and the classification task, the top-ranked approach involves combining a RF model with RFE. As a wrapper method, RFE iteratively selects features that maximize the ML model's performance by removing the least important features based on model feedback, thereby tailoring the feature set to the model's structure. In addition, in both tasks, the combination of an RF model with the VT method was found to rank among the highest-performing approaches. The VT method removes features with a variance below a specified threshold—in this case, a cutoff of 0.8, which is likely to exclude only sparse features or ASVs in metabarcoding datasets. Improving model performance by removing the “rare biosphere”, which is composed of sparsely distributed taxa, suggests that these taxa may have limited relevance for modeling ecological processes. However, this outcome may also be attributed to technical factors: RF decision trees are constructed by iteratively identifying splits that lead to subsets with increasingly homogeneous target values. Low-variance features, due to limited variability, provide few effective splits and are therefore less likely to be selected during tree construction. In addition, low-variance features may degrade RF performance due to the random selection of features for each tree. With a higher proportion of features that do not contribute to model performance, the probability of including relevant features in each tree decreases, potentially diminishing model accuracy. Consequently, rather than prioritizing highly relevant features, the VT method likely improves the RF performance by discarding features that contribute noise. This highlights the utility of VT in terms of reducing feature space, enhancing computational efficiency, and facilitating more consistent inclusion of informative features for RF models in high-dimensional metabarcoding datasets.

Given that the RF model without FS is the second-highest ranked approach for regression on both relative and absolute ASV counts, we can infer that most FS methods inadvertently degrade model performance by removing OTUs or ASVs relevant for the task at hand. For example, a linear FS method might remove features that correlate strongly with the target variable but do so non-linearly. This hypothesis is supported by our finding that for those datasets and target variables for which a linear FS method improves RF performance, other linear FS methods do the same, suggesting that the relationships being modeled in these cases are indeed linear (Fig. 4). The absence of a single consistently optimal FS method also indicates that we lack a function that can reliably distinguish ASVs that respond to or interact with an environmental target variable from ones that do not across diverse datasets and ecological contexts. Rather, RF and GB models are capable of flexibly approximating the complex ecological relationships inherent in metabarcoding data. Furthermore, tree-based ensemble methods are naturally equipped to handle high-dimensional data, leveraging their nonlinear and hierarchical structure to capture intricate patterns even with irrelevant features present. Such adaptability underscores the strength of RF and GB as robust modeling choices for metabarcoding applications, where ecological relationships are rarely straightforward and feature relevance may vary substantially across datasets. Note, however, that, on average across datasets and target variables, the RF and GB studied in this study achieve, at best, $R^{2}\approx 0.74$, leaving room for the improvement of machine learning models for microbial ecology.

Taken together, our results highlight the need for the development of ML methods that are explicitly compositionality-aware, just as have been developed for dimensionality reduction and beta-diversity analyses [84], [85]. Some progress has already been made in this direction, such as the introduction of balance trees, which can better handle compositional structures [86], [87], [88]. Nevertheless, standard RF models are currently more performant, interpretable, and accessible than compositional alternatives, indicating that compositionality-aware approaches are still in early development stages.

## 4 Conclusion

The high dimensionality inherent to data generated by techniques like metabarcoding necessitates effective FS strategies that improve the efficiency and interpretability of many data analysis methods. By systematically evaluating FS methods across 13 environmental metabarcoding datasets in a ML framework, our findings reveal that (i) ensemble models, particularly RF and GB, consistently outperform other models for regression tasks, (ii) RF and Gaussian Naive Bayes models yield the best performance in classification tasks, and (iii) coupling RFE with RF provides optimal results across most datasets and tasks. Notably, RF models without FS also ranked highly, thereby underscoring ensemble model robustness in high-dimensional settings. We expect these results to generalize to other environmental metabarcoding datasets. By openly sharing the framework developed for this benchmark, we can support further advances in FS methodologies specifically tailored for environmental metabarcoding datasets. Our modular and reproducible framework not only allows flexibility in testing various FS approaches but also enables the ML community to refine and adapt FS methods to better capture the complexities of ecological data. Our hope is that this contribution will drive further progress in FS and ML model performance for high-dimensional ecological datasets, ultimately enhancing the applicability of ML in environmental research.

## CRediT authorship contribution statement

**Erik Zschaubitz:** Writing – original draft, Validation, Software, Project administration, Methodology, Formal analysis, Data curation, Conceptualization. **Henning Schröder:** Writing – review & editing, Software, Resources, Methodology, Formal analysis, Data curation. **Conor Christopher Glackin:** Writing – review & editing. **Lukas Vogel:** Writing – review & editing. **Matthias Labrenz:** Writing – review & editing, Funding acquisition. **Theodor Sperlea:** Writing – original draft, Visualization, Validation, Supervision, Project administration, Methodology, Data curation, Conceptualization.

## Declaration of Competing Interest

The authors declare that they have no known competing financial interests or personal relationships that could have appeared to influence the work reported in this paper.

## Data Availability

The metabarcoding datasets used in this study are publicly available (details and weblinks are provided in Table S1). The code for the mbmbm framework as well as the R code used to select datasets and generate figures is available at https://github.com/erikzsch/mbmbm.

## Appendix A Supplementary material

MMC 1 Details on data sources and access methods for the datasets used in the study. MMC 1

MMC 2 Complete benchmark results for all analyses presented in this study. MMC 2

MMC 3 Statistics of the model comparison presented in Fig. 2. MMC 3

MMC 4 Complexity ranking of all datasets used in this study. Scatterplots representing key complexity metrics of microbial datasets, including the logarithm of the number of ASVs, the logarithm of the average variance of ASVs, and the dataset sparsity percentage. Each dataset was labeled on the vertical axis. MMC 4
