# Supplementary figures and images for: A benchmark analysis of feature selection and machine learning methods for environmental metabarcoding datasets
Source: Comput Struct Biotechnol J. 2025 Apr 16;27:1636–47. doi: 10.1016/j.csbj.2025.04.017 (PMC12049816; doi:10.1016/j.csbj.2025.04.017)

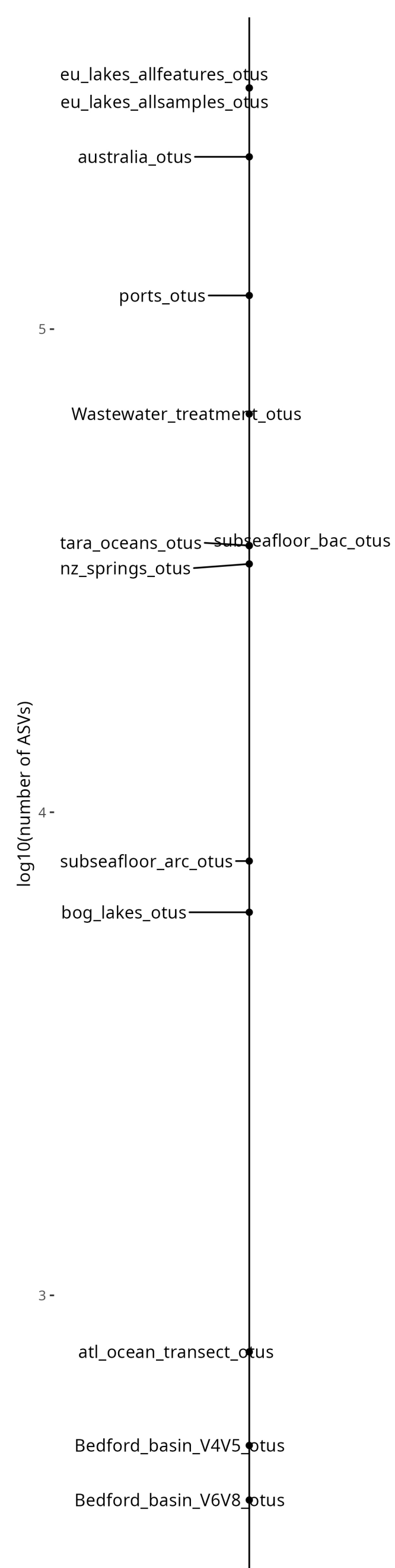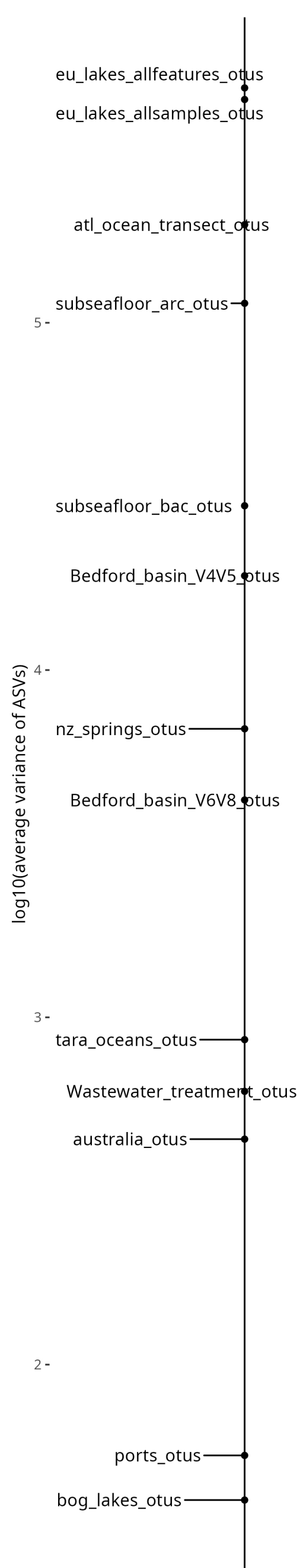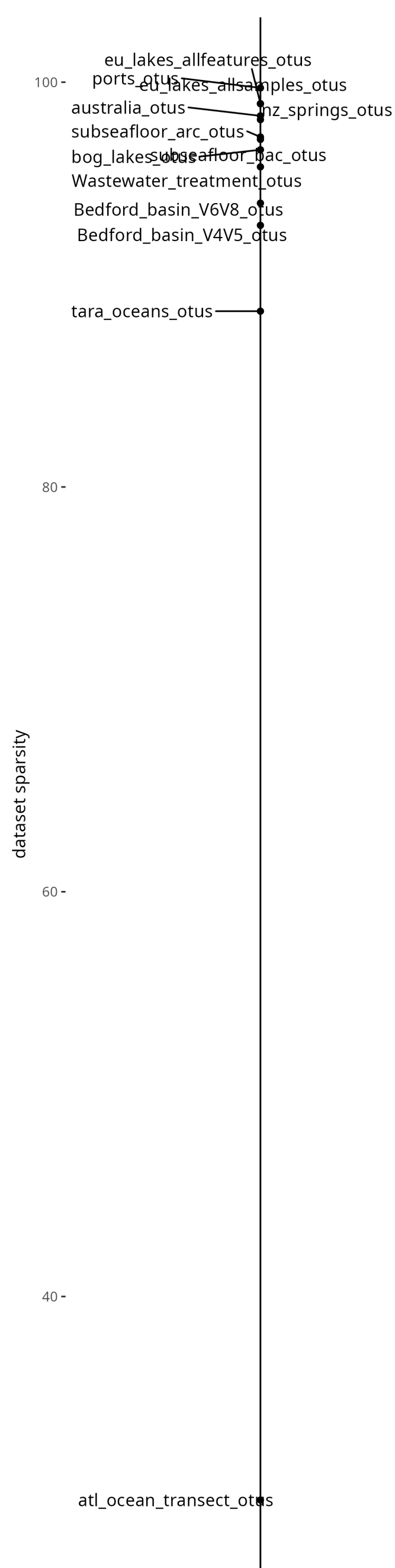

Supplement: MMC 4 — Complexity ranking of all datasets used in this study. Scatterplots representing key complexity metrics of microbial datasets, including the logarithm of the number of ASVs, the logarithm of the average variance of ASVs, and the dataset sparsity percentage. Each dataset was labeled on the vertical axis. [file mmc4.pdf]
